# Metastatic Ovarian Mucinous Carcinoma Presenting as Ileocecal Adenocarcinoma: A Case Report

**DOI:** 10.7759/cureus.24589

**Published:** 2022-04-29

**Authors:** Stephen E Glombicki, Diego Montelongo, Swethen Dushianthan, Camilo J Perez, Mario Cervantes, Alan P Glombicki

**Affiliations:** 1 Internal Medicine, University of Texas Health Science Center, Houston, USA; 2 Internal Medicine, Universidad Popular Autónoma del Estado de Puebla, Puebla, MEX; 3 Internal Medicine, Caribbean Medical University, Willemstad, CUW; 4 Obstetrics and Gynecology, Universidad Popular Autónoma del Estado de Puebla, Puebla, MEX; 5 Pathology, United Memorial Medical Center, Houston, USA; 6 Gastroenterology and Hepatology, Houston Digestive Diseases Consultants, Houston, USA

**Keywords:** immunohistochemistry, mucinous carcinoma, metastasis, ovarian cancer, colorectal cancer

## Abstract

Colorectal invasion is an unusual late-stage presentation of metastatic primary mucinous ovarian cancer. In this article, we report a fatal case of a 65-year-old female who presented to our clinic with progressive weight loss, severe constipation, and postprandial early satiety. She underwent an esophagogastroduodenoscopy (EGD) and colonoscopy. Direct visualization during colonoscopy revealed acute inflammation with ulceration and highly atypical glands in the ileocecal valve. The initial biopsy was unremarkable, and a repeat biopsy was performed due to high suspicion of malignancy. The repeat biopsy revealed poorly differentiated, invasive colon adenocarcinoma with partial mucinous features. The patient was referred to the surgery service. While planning for surgical resection, they obtained a CT abdomen and pelvis, which revealed a large ovarian mass and peritoneal carcinomatosis. Immunohistochemistry for the tumor cells was positive for pancytokeratin and cytokeratin 7, partially positive (up to 20%) for cytokeratin 20 and CDX2, and negative for estrogen receptors, monoclonal carcinoembryonic antigen (CEA), and synaptophysin. This immunophenotypic pattern is strongly consistent with metastatic mucinous carcinoma of ovarian origin.

## Introduction

Worldwide, colorectal cancer (CRC) is the second most common cancer in women. It is also the fourth most common cause of death from cancer, estimated to be responsible for almost 700,000 cancer deaths per year. The survival rate depends largely on the stage at diagnosis. The five-year survival rate is 90% for cases diagnosed at an early or localized stage (40% of cases), compared to 13% for those diagnosed at a late stage with distant metastasis (20% of cases). Australia and New Zealand have the highest estimated survival rates while Western Africa has reported the lowest. The patterns of colorectal cancer cases in men and women are similar globally [1].

Colorectal metastasis from ovarian carcinoma can present between one and 22 years after the initial diagnosis of primary ovarian cancer, with an average of nine years [2]. Symptoms such as a change in bowel habits, hematochezia, fatigue, abdominal pain, and unintended weight loss may be subtle or absent [3].

Mucinous ovarian carcinomas are a subgroup of epithelial ovarian cancer (EOC). They often present at an early stage (I-II) and have an overall five-year survival rate higher than 80%. However, the average survival time drops to 15 months when diagnosed at stage III [4]. The presence of multiple peritoneal implants (carcinomatosis) is independently correlated with reduced overall survival, and these patients are 2.3 times more likely to die when compared to patients with other subtypes of EOC [5].

In this paper, we discuss a case of fatal primary ovarian mucinous carcinoma with colonic metastases, carcinomatosis, and diffuse omental invasion. The ileocecal ulcerated mass was initially suspected to be of primary colonic origin.

## Case presentation

A 65-year-old female with a history of osteoarthritis, pre-diabetes, hypercholesterolemia, and constipation-predominant irritable bowel syndrome (IBS-C) presented to the gastroenterology specialty clinic for progressive weight loss (more than 21 pounds in the past four months), severe constipation, dyspepsia, and early satiety. She denied taking prescription medications and denied the use of alcohol, tobacco, or illicit drugs. Previous screening with a fecal occult blood test (FOBT) was negative. An EGD and colonoscopy were performed.

Pathological results of the EGD revealed partial goblet cell intestinal metaplasia and fundic gland polypoid change, mild reflux esophagitis at the squamocolumnar boundary (gastroesophageal junction), and healing chemical gastropathy changes in the antrum.

**Figure 1:**
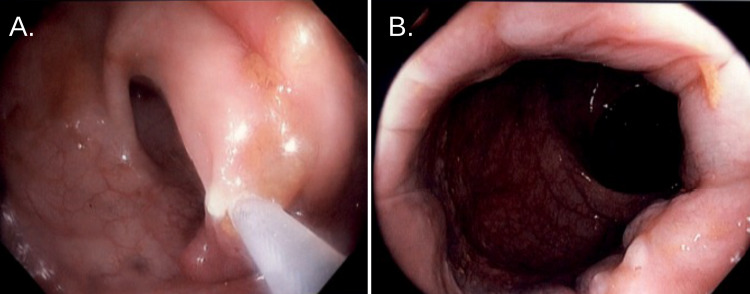
Colonoscopy, noting an appendiceal orifice and ileocecal valve A. At the edge of the ileocecal valve, there was ileocecal colitis erythema, edema, and friability. B. Tissue void after multiple biopsies were taken for histopathology.

Biopsies were obtained during colonoscopy. Pathological results revealed tubular adenomas in the rectum and transverse colon, retention type changes in the sigmoid, and acute inflammation with ulceration and highly atypical glands in the ileocecal valve (Figure 1).

**Figure 2:**
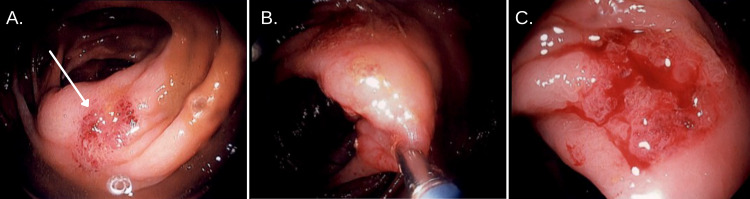
Second colonoscopy A. A 2-cm erythematous friable sessile plaque was noted on the cephalad margins of the ileocecal valve (consistent with a 0-IIa type lesion-Paris classification). B. Biopsy instrumentation, continued friable erythema of ileocecal valve. C. Sessile plaque after several biopsies were taken. Moderate friability.

The tissue specimen obtained during colonoscopy required a repeat biopsy in order to exclude malignancy. A second colonoscopy confirmed the initial suspicion of poorly differentiated, invasive colon adenocarcinoma with partial mucinous features (Figure 2).

**Figure 3:**
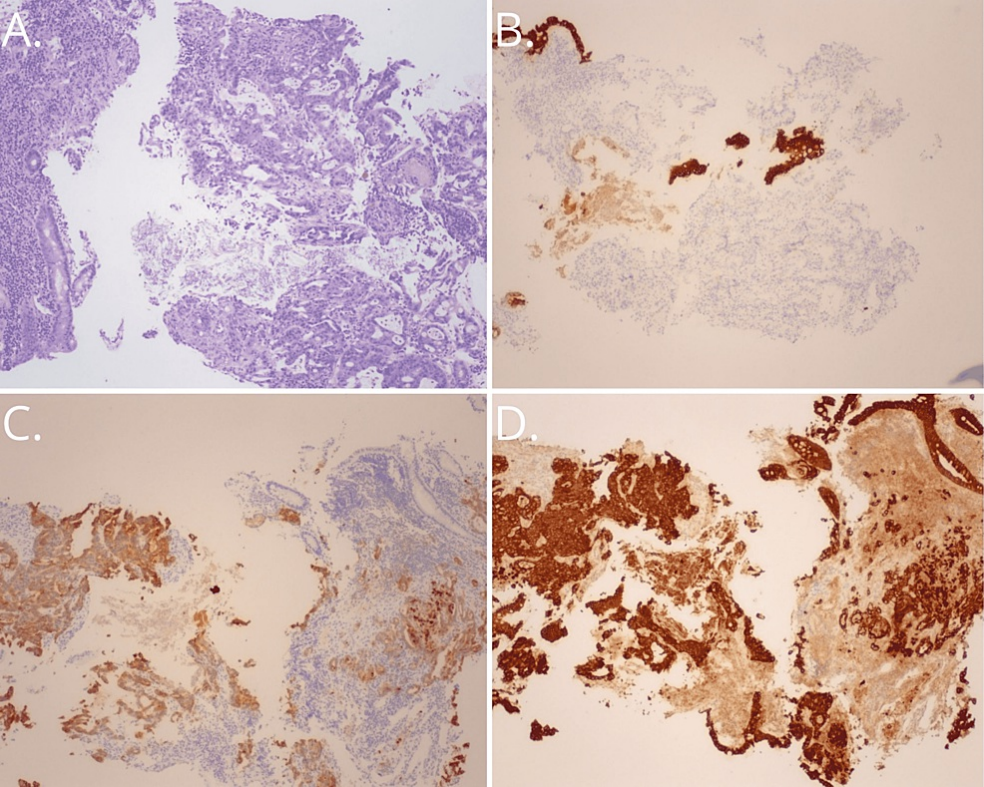
Ileocecal valve specimen revealed positivity for pancytokeratin, diffuse expression of CK7, and partial positivity for CK20 and CDX2; 4X magnification (4x) A. H&E, rebiopsy showing poorly differentiated, invasive colon carcinoma with partial mucinous features (4x). B. CK20 is negative in cancer glands and residual colonic glands are positive (4x). C. CK7 positive - reverse pattern, tumor showing positive features, and residual colonic glands are negative (4x). D. Pancytokeratin positive for both normal colonic glands and tumor glands (4x).

The patient was referred to the surgery service for a resection evaluation. In preparation for the operation, the surgery team's management included imaging, which revealed a large ovarian mass and peritoneal carcinomatosis. At this time, the pathology report was amended to include specific immunohistochemistry (IHC) assays. These described the mucinous features of the uncommon ileocecal ulcer. The tumor cells were positive for pancytokeratin and had a diffuse expression of cytokeratin 7 (CK7). They were partially positive (up to 20%) for CK20 and CDX2 but negative for estrogen receptors (ER), monoclonal carcinoembryonic antigen (CEA), and synaptophysin. This immunophenotypic pattern is strongly consistent with a metastatic mucinous ovarian carcinoma (Figure 3).

An exploratory laparotomy with right hemicolectomy and left oophorectomy was performed, and omental and peritoneal biopsies were taken. Biopsies revealed multiple peritoneal and pericolonic fat tumor foci (carcinomatosis). The excisioned serosal wall tumor mass measured 4.2 x 3 cm. There was also ileocecal valve ulceration, large omental nodules (consistent with metastatic mucinous carcinoma), as well as a left ovarian mass that measured 6.7 x 5 x 4 cm, consistent with mucinous invasive carcinoma.

The patient deteriorated shortly after arriving at the recovery room so a repeat laparotomy was performed. No overt bleeding was found, and minimal serous fluid and venous blood were aspirated. Postoperatively, she returned to the hospital's surgical unit. The patient’s condition rapidly deteriorated, and despite resuscitative efforts, she expired.

## Discussion

Ovarian cancer is the eighteenth most common cancer worldwide and the seventh most common cancer in women. Nearly 313,959 new cases and 207,252 new deaths from ovarian cancer were reported in 2020 [6]. Since the early stages have no symptoms, ovarian cancer is often diagnosed at a late stage. The five-year survival rate ranges from 30% to 50% [7]. Ovarian carcinoma often invades the contralateral ovary, pelvis, omentum, and peritoneum. In only 4%-6% of the cases, primary ovarian cancer metastasizes to the colon. When found, its usual location is the sigmoid colon or rectum. Ileocecal involvement is quite rare [8].

Primary ovarian cancers can spread to the gastrointestinal tract by four different routes: lymphogenous, hematogenous, direct infiltration of the bowel wall, and transcoelomic; transcoelomic is the main form of colonic involvement [9].

To date, only one case report details cecal metastasis, and it was found 24 years after resection of the primary ovarian tumor [10]. In contradistinction, the current case report stands in line with a rapid deterioration from metastatic ovarian carcinoma.

Generally, the macroscopic features of tumors are best assessed via direct visualization, during endoscopy and laparotomy. Imaging can also help assess size and laterality. However, these techniques are insufficient to differentiate primary from metastatic ovarian mucinous tumors. Therefore, microscopic features and IHC assays can help classify tumors that have invaded the lower gastrointestinal tract [11]. Primary mucinous ovarian tumors express CK7 in 90% of the cases, mostly in a diffuse pattern, while CK20 is expressed in 65%-70%. CK7+/CK20+ has been observed in 67%-74% [5,10-12], CK7+/CK20- in 26%, and CK7-/CK20+ in 7% of cases [11,13]. CDX2 positivity is found in 49%-64% of primary mucinous ovarian tumors; however, its expression has also been found in metastatic appendiceal, pancreaticobiliary, and gastric carcinomas [11-14]. Therefore, when positive, it should be compared with markers like monoclonal CEA, ER, and synaptophysin, in conjunction with CK7 and CK20, in order to narrow the differential.

## Conclusions

Secondary mucinous carcinoma of the ileocecal valve from a primary ovarian carcinoma is extremely rare. The clinician needs to differentiate this challenging case from a primary colonic carcinoma. To recognize metastasis, the treatment team needs a high index of suspicion, thorough physical examination, careful attention to lesion location on imaging studies and during colonoscopy, as well as IHC assays (when available). These are the cornerstones of accurate diagnosis.
